# Human acid-sensing ion channel 1a/3 heteromers at a 1:2 ratio expand the functional capabilities of homomeric ASIC3 and are likely to be physiologically relevant

**DOI:** 10.1093/nsr/nwaf418

**Published:** 2025-10-07

**Authors:** Dmitry I Osmakov, Elisaveta S Dubodel, Aleksandr P Kalinovskii, Sergey G Koshelev, Yaroslav A Andreev, Yuliya V Korolkova, Sergey A Kozlov

**Affiliations:** Shemyakin–Ovchinnikov Institute of Bioorganic Chemistry, Russian Academy of Sciences, Russia; Shemyakin–Ovchinnikov Institute of Bioorganic Chemistry, Russian Academy of Sciences, Russia; Moscow Center for Advanced Studies, Russia; Shemyakin–Ovchinnikov Institute of Bioorganic Chemistry, Russian Academy of Sciences, Russia; Shemyakin–Ovchinnikov Institute of Bioorganic Chemistry, Russian Academy of Sciences, Russia; Shemyakin–Ovchinnikov Institute of Bioorganic Chemistry, Russian Academy of Sciences, Russia; Shemyakin–Ovchinnikov Institute of Bioorganic Chemistry, Russian Academy of Sciences, Russia; Shemyakin–Ovchinnikov Institute of Bioorganic Chemistry, Russian Academy of Sciences, Russia

Acid-sensing ion channels (ASICs) are proton-gated cation channels that belong to the degenerin–epithelial sodium channel (DEG/ENaC) family. In mammals, at least six subunits (ASIC1a, ASIC1b, ASIC2a, ASIC2b, ASIC3 and ASIC4) have been identified, forming homo- and heterotrimeric complexes with distinct proton sensitivity, channel kinetics and responsiveness to various positive and negative modulators. Among them, ASIC1a and ASIC3 are particularly significant due to their high pH sensitivity. ASIC3 channels exhibit faster desensitization kinetics and are capable of generating a sustained current component that follows the transient component [1,2]. ASICs are expressed in both central and peripheral sensory neurons, where they are implicated in various physiological and pathological processes, such as synaptic plasticity, fear conditioning, learning, memory, ischemic stroke, neurodegeneration, nociception, inflammatory processes, tumor development, mechanical hyperalgesia and myocardial ischemia [3,4].

Despite the significant physiological roles of ASIC3 channels, which have been predominantly studied in rodent models, their human orthologs remain largely underexplored, particularly regarding their heteromeric assembly with other isoforms and their precise composition in the human peripheral nervous system. This gap in knowledge is especially important given that, unlike in rodents, in which ASIC3 channels are primarily expressed in peripheral sensory neurons [3], in humans, they are widely distributed in both the peripheral and central nervous systems [5,6] (https://www.proteinatlas.org/ENSG00000213199-ASIC3). A particularly intriguing functional feature unique to homomeric human ASIC3 (hASIC3) channels is their pronounced steady-state desensitization (SSD) under physiological conditions (pH 7.3–7.4). In other words, when activated by acidification from a conditioning pH of 7.4, hASIC3 exhibits transient currents of significantly lower amplitude compared with those observed at more alkaline (and less physiological) pH values (≥8.0) [7,8]. This phenomenon suggests that homomeric channels transition into a desensitized state without prior activation, which may limit their function as homomers under physiological conditions. We have previously hypothesized that one possible mechanism enabling full functionality of homomeric hASIC3 under physiological conditions involves interactions with endogenous ligands, such as isoquinoline alkaloids, which have been shown to prevent desensitization and restore the transient current component [8].

In this study, we propose an alternative explanation: homomeric ASIC3 is likely not abundant in the peripheral nervous system (PNS) and primarily functions within heteromeric channels. Given that human ASIC1a is also abundantly expressed in the PNS [9], this study focuses on characterizing ASIC1a/ASIC3 heteromers. Through the functional characterization of heteromers formed by co-expressing ASIC1a and ASIC3 in different ratios and generating concatemeric constructs with defined stoichiometries, we provide evidence that ASIC1a/ASIC3 heteromers function more effectively at physiological pH than homomeric hASIC3, with the 1a–3–3 concatemer closely matching the properties of native heteromers.

We expressed human ASIC channels in *Xenopus laevis* oocytes and characterized their properties by using two-electrode voltage clamp electrophysiology. The study examined the SSD and activation properties by determining the pH values for half-maximal SSD (pH₅₀SSD) and activation (pH₅₀act), Hill coefficients and desensitization time constants (τ_des_). Homomeric hASIC1a exhibited inward currents with a pH₅₀SSD of 7.14 ± 0.01 and pH₅₀act of 6.59 ± 0.02 (Fig. 1A–E and Figs S1 and S2). The τ_des_ at pH 5.5 activation was 1.52 ± 0.21 s (Fig. S2). Homomeric hASIC3, however, generated only 8% ± 2% of its maximal current at pH 7.4, with a pH₅₀SSD of 7.64 ± 0.01 and pH₅₀act of 6.39 ± 0.01 (Fig. 1A–E and Fig. S1), showing a significant alkaline shift in the SSD and an acidic shift in activation compared with hASIC1a. The τ_des_ for hASIC3 was 0.27 ± 0.03 s (Fig. S2), reflecting its rapid desensitization kinetics.

**Figure 1.**
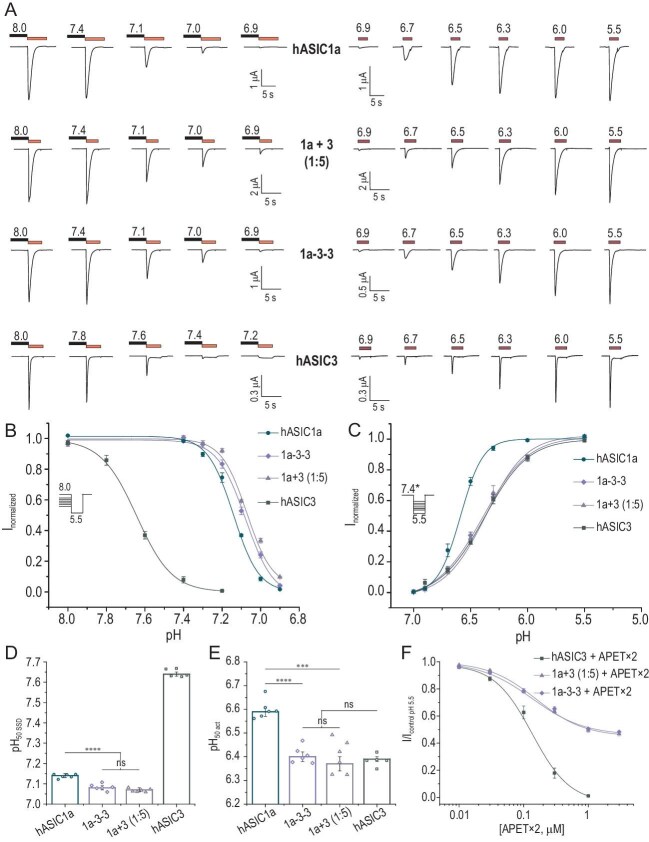
The heteromeric 1a+3 (1:5) channel and the concatemeric 1a-3–3 channel exhibit similar functional characteristics. (A) Representative current traces for steady-state desensitization (SSD, left panel) and activation (right panel) of the indicated channels. SSD traces were recorded from the same oocyte, with activation by a pH 5.5 stimulus following pre-incubation at various conditioning pH levels. Activation traces were also recorded from the same cell, with stimulation from a conditioning pH of 7.4, except for hASIC3, which had a conditioning pH of 8.0. hASIC1a and hASIC3 represent wild-type human homomeric ASIC1a and ASIC3 channels, respectively. The heteromeric 1a+3 (1:5) channel corresponds to oocytes co-expressing hASIC1a and hASIC3 at a 1:5 ratio. (B, C) pH dependence curves for (B) SSD and (C) activation for the same channels, fitted with Hill function (solid lines). In (C), the asterisk indicates that the pH dependence of activation was determined for all channels at a conditioning pH of 7.4, except for hASIC3, for which the conditioning pH was 8.0. Each point represents data from five or six cells. (D, E) Bar plots of individual pH₅₀ values for (D) SSD and (E) activation for each channel type. Data are presented as mean ± SEM. Statistical significance: ***P < 0.001, ****P < 0.0001, ns (not significant); one-way ANOVA followed by Dunnett’s post hoc test. (F) Dose–response curves for the inhibitory effect of APETx2 on hASIC3 (*n* = 5), 1a+3 (1:5) (*n* = 10) and 1a-3–3 (*n* = 5). The peptide inhibitor APETx2 was pre-incubated for 30 s before activation with a pH 5.5 stimulus. Data are presented as mean ± SEM.

To study ASIC1a/3 heteromers, we injected hASIC1a and hASIC3 messenger ribonucleic acid (mRNA) into oocytes at 5:1 and 1:5 ratios. Under activation conditions, heteromers exhibited similar behavior to those of their predominant subunits, with pH₅₀act values of 6.56 ± 0.02 (5:1) and 6.37 ± 0.03 (1:5) (Fig. 1A–E and Figs S1 and S2). However, the SSD properties differed significantly from those of homomeric channels. Both heteromers exhibited a shift toward more acidic SSD values (pH₅₀SSD of 7.01 ± 0.01 and 7.07 ± 0.01 for 5:1 and 1:5, respectively), enabling channel function at physiological pH. The τ_des_ values for 1a+3 (5:1) remained similar to those of hASIC1a, whereas 1a+3 (1:5) had an intermediate τ_des_ of 0.80 ± 0.04 s, which was significantly different from those of both homomeric counterparts (Fig. S2). These results indicate that the incorporation of the ASIC1a subunit into an ASIC3-containing complex restores its functionality at physiological pH.

To confirm these findings, we generated concatemeric constructs linking three ASIC subunits in defined arrangements by using linker sequences (Fig. S3). Eight concatemers were created, including 1a–1a–1a, 3–3–3, 1a–1a–3, 1a–3–1a, 3–1a–1a, 3–3–1a, 3–1a–3 and 1a–3–3. Functional characterization revealed that the 3–3–3 concatemer exhibited properties that were identical to those of homomeric hASIC3, with pH₅₀SSD of 7.63 ± 0.01, pH₅₀act of 6.41 ± 0.03 and τ_des_ of 0.28 ± 0.02 s (Figs S4 and S5), confirming its desensitization at physiological pH. Replacing one ASIC3 subunit in the 3–3–3 concatemer with ASIC1a (1a–3–3 or 3–3–1a) altered the channel properties. Both 1a–3–3 and 3–3–1a no longer desensitized at pH 7.4, with pH₅₀SSD values of 7.08 ± 0.01 and 7.21 ± 0.01, respectively—significantly more acidic than 3–3–3 (Figs S4 and S5). The pH₅₀act of 1a–3–3 remained similar to that of hASIC3 (6.4 ± 0.02) (Fig. 1C and E), while 3–3–1a showed an alkaline shift (6.54 ± 0.01) (Figs S4 and S5). The τ_des_ values were also significantly increased (0.61 ± 0.05 s for 1a–3–3, 0.44 ± 0.04 s for 3–3–1a) (Fig. S5), indicating reduced desensitization. These findings confirm that a single ASIC1a subunit in an ASIC3-dominant complex is sufficient to enable function at physiological pH.

Heteromeric channel 1a+3 (1:5) and the 1a–3–3 concatemer exhibit nearly identical functional properties, suggesting that, in 1a+3 (1:5), subunit 3 predominates. Their key parameters, including pH₅₀SSD, pH₅₀act and Hill coefficients, show no significant differences (Fig. 1D and E, and Table S1), except for τ_des_ (Fig. S6), which is likely influenced by the absence of free N- and C-termini. In contrast, the 3–3–1a concatemer differs significantly from 1a+3 (1:5) in all key parameters (Fig. S6 and Table S1), indicating the crucial role of the ASIC1a subunit’s free N-terminus in pH-dependent SSD and activation. Pharmacological analysis with APETx2, a known ASIC3 inhibitor, further supports the similarity between 1a+3 (1:5) and 1a–3–3 (Fig. 1F). APETx2 inhibits hASIC3 and 3–3–3 channels with similar efficacy, showing IC₅₀ values of 135 ± 18 and 129 ± 13 nM, respectively (Fig. S7). When tested on heteromeric 1a+3 (1:5) and the concatemers, APETx2 only partially inhibited currents at pH 5.5, even at high concentrations (3 µM). The saturation of APETx2’s inhibitory effect was identical for 1a+3 (1:5) and 1a–3–3, at 55% ± 2% (IC₅₀ 157 ± 17 nM) and 54% ± 2% (IC₅₀ 135 ± 23 nM), respectively, while, for 3–3–1a, it was 66% ± 1% (IC₅₀ 133 ± 10 nM) (Fig. 1F and Fig. S7). These results indicate that 1a+3 (1:5) and 1a–3–3 are functionally equivalent in their ligand interactions.

It is important to acknowledge certain limitations associated with *Xenopus* oocytes as an expression system, particularly regarding the lipid composition of the membrane in which the ASIC channels are embedded. Notably, a recent study demonstrated that ASIC3 function is strongly regulated by lipids, with polyunsaturated fatty acids relieving the resting block [10]. This lipid dependence may influence the physiological behavior of ASIC3-containing heteromers. Although, as noted above, the substantial desensitization of homomeric ASIC3 at pH 7.4 has also been observed in CV-1 in Origin with SV40 genes (COS) cells [7], further studies in mammalian expression systems are necessary to validate our findings regarding the differences in behavior between human homomeric ASIC3 and ASIC1a/3 heteromers.

Nevertheless, our study suggests that, in the human nervous system, ASIC3 channels primarily exist as heteromers with ASIC1a. This is supported by ribonucleic acid (RNA) scope data showing that 43% of human primary sensory neurons—and, notably, 66% of TRPV1-positive neurons—co-express ASIC1 and ASIC3, whereas only 1.5%–2% express ASIC3 alone [9]. These findings underscore the physiological relevance of ASIC1a/3 heteromers. Notably, heteromeric ASIC1a/ASIC3 channels, particularly at a 1:2 stoichiometry—evidenced by the shared key properties between co-expressed ASIC1a and ASIC3 subunits and the 1a–3–3 concatemer—maintain full functionality under physiological conditions.

Overall, our study advances the understanding of ASIC channel structure and pharmacology, reinforcing the 1a–3–3 concatemer as a relevant model for drug development targeting ASIC3-related pathologies. Additionally, these findings have significant implications for pain research, as targeting specific ASIC1a/3 heteromers may facilitate the development of novel analgesics. Given the established role of ASIC3 in inflammatory pain, postoperative pain and myocardial ischemia, it represents a promising target for therapeutic intervention. By identifying the precise subunit composition necessary for ASIC3 function in human neurons, our study provides critical insights into ASIC pharmacology and lays the groundwork for designing more selective ASIC inhibitors or modulators for pain relief.

## Supplementary Material

nwaf418_Supplemental_File
