# Supplementary material for: Human acid-sensing ion channel 1a/3 heteromers at a 1:2 ratio expand the functional capabilities of homomeric ASIC3 and are likely to be physiologically relevant
Source: Natl Sci Rev. 2025 Oct 7;12(11):nwaf418. doi: 10.1093/nsr/nwaf418 (PMC12617345; doi:10.1093/nsr/nwaf418)
Supplement: nwaf418_Supplemental_File [file nwaf418_supplemental_file.doc]

# Supplementary materials for

**Human Acid-Sensing Ion Channel (ASIC) 1a/3 Heteromers in a 1:2 Ratio Expand the Functional Capabilities of Homomeric ASIC3 and Are Likely to Be Physiologically Relevant**

Dmitry I. Osmakov^1,*^, Elisaveta S. Dubodel^1,2^, Aleksandr P. Kalinovskii^1^, Sergey G. Koshelev^1^, Yaroslav A. Andreev^1^, Yuliya V. Korolkova^1^, Sergey A. Kozlov^1^

^1^Shemyakin–Ovchinnikov Institute of Bioorganic Chemistry, Russian Academy of Sciences, Ul. Miklukho-Maklaya 16/10, 117997 Moscow, Russia

^2^Moscow Center for Advanced Studies, Kulakova Str. 20, 123592 Moscow, Russia

^*^Correspondence: [serg@ibch.ru](mailto:serg@ibch.ru)

**This** **file includes:**

Text S1

Figures S1 to S7

Table S1

## Text S1 Materials and methods

Human ASIC1a and ASIC3 cDNAs

Human ASIC1a cDNA was cloned from the total cDNA of SH-SY5Y cells differentiated by all-trans-retinoic acid. SH-SY5Y were cultivated and differentiated as described elsewhere [1]. Total RNA from the cells was extracted using TRIzol Reagent (Life Technologies, Carlsbad, CA, USA), chloroform phase separation, and ethanol precipitation at −20 ⁰C. First-strand cDNA was synthesized using Mint Kit (Evrogen, Moscow, Russia) according to the manufacturer’s guidelines and later used as a template for PCR. The primers were as follows: forward, ATATAGCTAGCCACCATGGAACTGAAGGCCGAG (containing a Kozak motif and NheI restriction site); reverse, TTGGATCCTCAGCAGGTAAAGTCCTCGAACG (containing a stop-codon and BamHI restriction site). The primers flank transcript variant 2 (NCBI GeneBank NM_001095) that encodes canonic isoform 2, or simply ASIC1a (1584 bp). PCR was conducted using Encyclo polymerase (Evrogen) under the following cycling parameters: initial denaturation 95 ⁰C for 1 min; 95 ⁰C – 20 s, 65 ⁰C – 20 s, 72 ⁰C – 2 min for 35 cycles; and final extension for 3 min. The amplicon of correct length (≈ 1.6 kbp) was subcloned into pVax1 vector (Invitrogen, Carlsbad, CA, USA) and confirmed using Sanger sequencing. Human ASIC3 cDNA was prepared as described [2].

Generation of Concatemeric ASIC Constructs

The plasmid vector pVax1 (Thermo Fisher Scientific, Waltham, MA, USA) was mutated using the Phusion Site-Directed Mutagenesis Kit (Thermo Fisher Scientific, Waltham, MA, USA) following the manufacturer's protocol. The primers used for mutagenesis were pVax_BSH_rev (5'-CGAGATCTAACGCTAGCCAGCTTGG-3') and pVax_BSH_frw (5'-TGGTCGACACATAAGCTTGGTACCGAGCT-3'). The final mutated vector, pVax1(BSH), was confirmed through DNA sequencing and subsequently used for cloning the concatemers.

The hASIC1a and hASIC3 genes were amplified using specific primer pairs for the first, second, and third subunits (Table 2), cloned into a TA vector (Eurogen, Moscow, Russia), and sequenced. The verified sequences of the individual subunits were then excised using appropriate restriction enzymes and sequentially ligated into the pVax1(BSH) vector, which had been linearized at the corresponding restriction sites. Intermediate target clones were selected using restriction analysis and PCR methods.

****Table 2.**** Primers used for cloning ASIC1a-3 concatemers. Restriction sites are in bold.

| **Subunit** | **Primers name** | **Primers structure** |
| --- | --- | --- |
| **hASIC3** | | |
| 3-1 | hA3-BglII-F1  hA3-SalI-R1 | 5’-ctcctt**agatct**atgaagcccacctcaggcc-3’  5’-gat**gtcgac**attattgagctgtgtgacaaggtagcag-3’ |
| 3-2 | hA3-SalI-F2  hA3-HindIII-R2 | 5’-aat**gtcgac**atcaataatatgaagcccacctcaggcc-3’  5’-tta**aagctt**gattattgagctgtgtgacaaggtagcag-3’ |
| 3-3 | hA3-HindIII-F3  hA3-EcoRI-R3 | 5’-atc**aagctt**taaataatatgaagcccacctcaggcc-3’  5’-att**gaattc**ctagagctgtgtgacaaggtagcag-3’ |
| **hASIC1a** | | |
| 1-1 | hA1a-BglII-F1  hA1a-SalI-R1 | 5’-ctcctt**agatct**atggaactgaaggccgaggag-3’  5’-gat**gtcgac**attattgcaggtaaagtcctcgaacgtgc-3’ |
| 1-2 | hA1a-SalI-F2  hA1a-HindIII-R2 | 5’-aat**gtcgac**atcaataatatggaactgaaggccgaggag-3’  5’-tta**aagctt**gattattgcaggtaaagtcctcgaacgtgc-3’ |
| 1-3 | hA1a-HindIII-F3  hA1a-EcoRI-R3 | 5’-atc**aagctt**taaataatatggaactgaaggccgaggag-3’  5’-att**gaattc**tcagcaggtaaagtcctcgaacgtgc-3’ |

Isolation of Xenopus laevis oocytes and mRNA Injection

Unfertilized oocytes were harvested from adult female Xenopus laevis frogs, maintained at 20 ± 2°C under a 10 h light/14 h dark cycle. Frogs were anesthetized using a 0.17% solution of tricaine methanesulfonate (MS222), and a small portion of the ovary was surgically removed through a minor incision in the abdomen. The incision was sutured, and the frogs were placed in a separate recovery tank until fully regaining consciousness. No signs of post-operative distress were observed. A minimum of three months was allowed between surgeries for each frog. The ND96 medium (96 mM NaCl, 2 mM KCl, 1.8 mM CaCl₂, 1 mM MgCl₂, and 5 mM HEPES, pH adjusted to 7.4 with NaOH) was used as the working solution. To remove the connective tissue and follicular layers, oocytes were incubated with 1 mg/mL collagenase in calcium-free ND96 medium for 2–3 hours at room temperature. Stage IV and V oocytes were then selected, sorted, and stored in ND96 medium. Approximately 16 to 18 hours after isolation, oocytes were injected with mRNAs using the Nanoliter 2000 microinjection system (World Precision Instruments, Sarasota, FL, USA). The mRNAs were synthesized from the pVAX1 plasmid encoding human ASIC1a and ASIC3, and pVAX1 (BSH) plasmid encoding ASIC1a/ASIC3 concatemers. The amounts of mRNA injected per oocyte were 0.2–0.5 ng for human ASIC1a, and 2.5–10 ng for human ASIC3 and concatemers. Following injection, oocytes were maintained at 18°C for 2–3 days, and subsequently stored at 15°C in ND96 medium supplemented with gentamicin (50 µg/mL) for up to 7 days.

Electrophysiological Recordings

Whole-cell ASIC currents were recorded using the two-electrode voltage clamp technique with a GeneClamp 500 amplifier (Axon Instruments, Union City, CA, USA), holding the membrane potential at –50 mV. Microelectrodes were filled with a 3 M KCl solution. The external bath solutions were ND96 (pH 7.6–7.3) or ND96 in which 5 mM HEPES was replaced by 5 mM HEPPS (pH 8.0 and 7.8) or by 5 mM MOPS (pH 7.2–6.7). The activating solution was ND96, in which 5 mM HEPES was substituted with either 10 mM MOPS (adjusted to pH 7.0, 6.9, and 6.7) or 10 mM MES (adjusted to pH 6.5, 6.3, 6.0, and 5.5). Solution flow rate and rapid exchange in the recording chamber (10 ml/min) were controlled using an in-house computer-operated valve system. Data were filtered at 10 Hz and digitized at 100 Hz using an L780M ADC (LCard, Moscow, Russia).

Data Analysis

Electrophysiological data analysis and visualization were performed using OriginPro 8.6.0 (OriginLab, Washington, DC, USA). The pH dependencies of steady-state desensitization and activation currents were fitted using the Hill equation:

F1(x) = ((A1 − A2)/(1 + (x/[H+]50)ˆnH)) + A2, where A1 is the minimum response value, A2 is the maximum response value, [H+]₅₀ is the proton concentration at which the current amplitude reaches half of its maximum value (Imax), and nH is the Hill coefficient. Imax was calculated from the amplitude values obtained at a given conditioning or activating pH for each cell by individual fitting. The data were then normalized to the calculated Imax value. The normalized data were averaged and fitted to the logistic equation F1(x).

Dose–response data for the inhibitory effect of APETx2 were fitted with the Hill equation:

Ix = I0/[1 + ([x0]/[x])ˆnH], where Ix is the current amplitude at a given concentration of the peptide ligand [x], I0 is the current amplitude in the absence of the peptide, and x₀ is the concentration at which APETx2 exhibits half of its maximal effect.

The rate of current decay was fitted for each individual experiment using a single exponential decay model:

F2(x) = A*eˆ(−x/τdes) + A0, where A0 is the baseline current amplitude, and τdes is the time constant of exponential current decay.

Data are presented as means ± SEM. Differences between groups were assessed using an unpaired t-test, with p < 0.05 considered statistically significant.

## Ethical statement

This study was conducted in strict compliance with the World Health Organization’s International Guiding Principles for Biomedical Research Involving Animals. The experimental protocol was approved by the Institutional Policy on the Use of Laboratory Animals at the Shemyakin–Ovchinnikov Institute of Bioorganic Chemistry, Russian Academy of Sciences (Protocol Number: 351/2022; approved on 24 November 2022). All procedures followed the ARRIVE (Animal Research: Reporting of In Vivo Experiments) guidelines and the European Convention for the Protection of Vertebrate Animals Used for Experimental and Other Scientific Purposes (Strasbourg, 18 March 1986).

## References

1. Kalinovskii AP, Osmakov DI, Koshelev SG, *et al.* Retinoic acid-differentiated neuroblastoma SH-SY5Y is an accessible in vitro model to study native human acid-sensing Ion channels 1a (ASIC1a). *Biology (Basel)* 2022; **11**: 167.

2. Osmakov DI, Koshelev SG, Andreev YA, *et al.* Proton-independent activation of acid-sensing ion channel 3 by an alkaloid, lindoldhamine, from Laurus nobilis. *Br J Pharmacol* 2018; **175**: 924–37.

## Figures


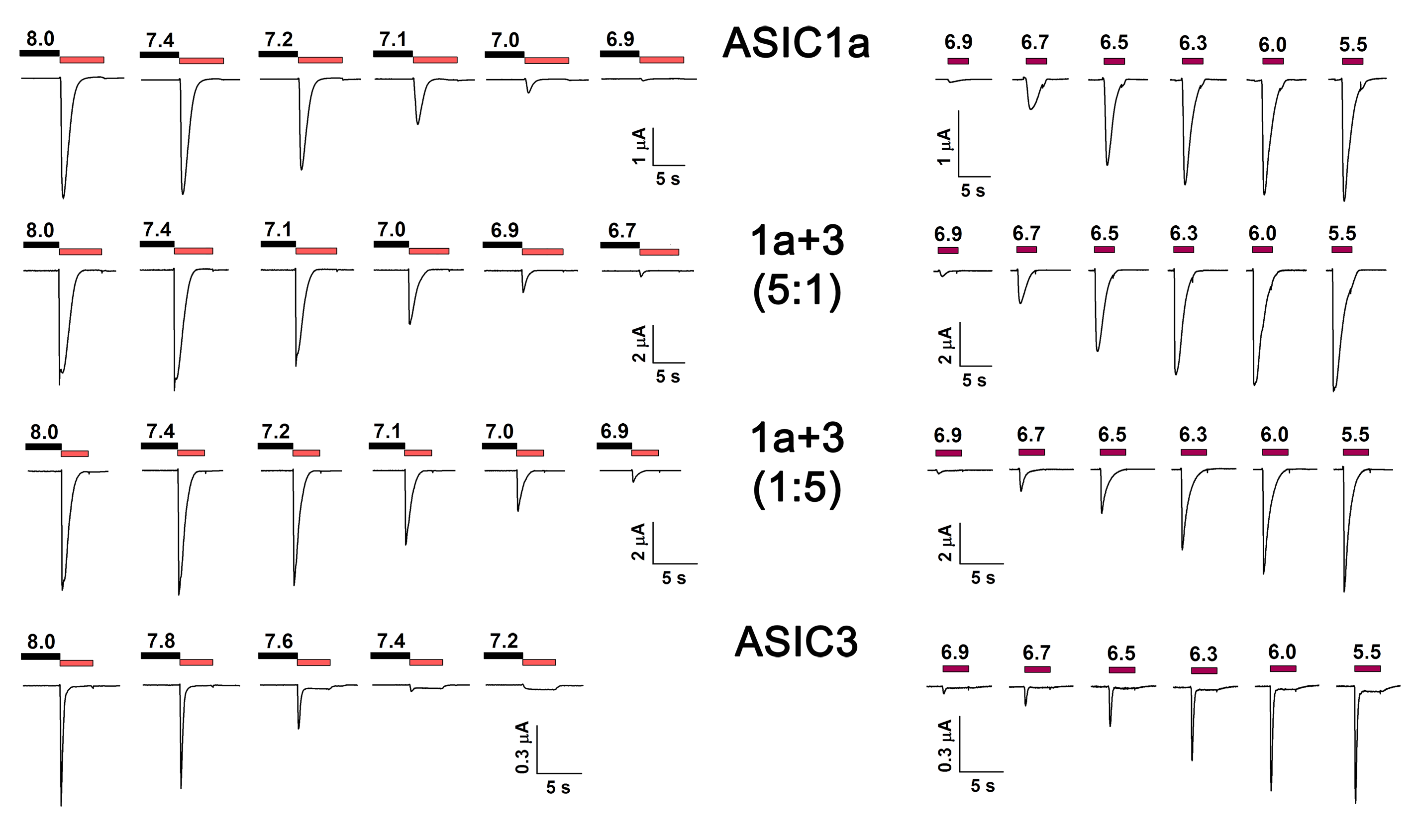


**Figure S1. pH dependence of steady-state desensitization (SSD) and activation for human ASIC1a (hASIC1a), human ASIC3 (hASIC3), and ASIC1a/3 heteromers.** Representative current traces for SSD (left panel) were recorded from the same cell, showing activation by a pH 5.5 stimulus (red bar) following pre-incubation at various conditioning pH levels. Representative current traces for activation were also recorded from the same cell, with activation occurring from a conditioning pH of 7.4 (except for ASIC3, where the conditioning pH was 8.0), followed by stimulation with different pH values. The heteromers 1a+3 (5:1) and 1a+3 (1:5) represent oocytes co-expressing hASIC1a and hASIC3 mRNA at ratios of 5:1 and 1:5, respectively.


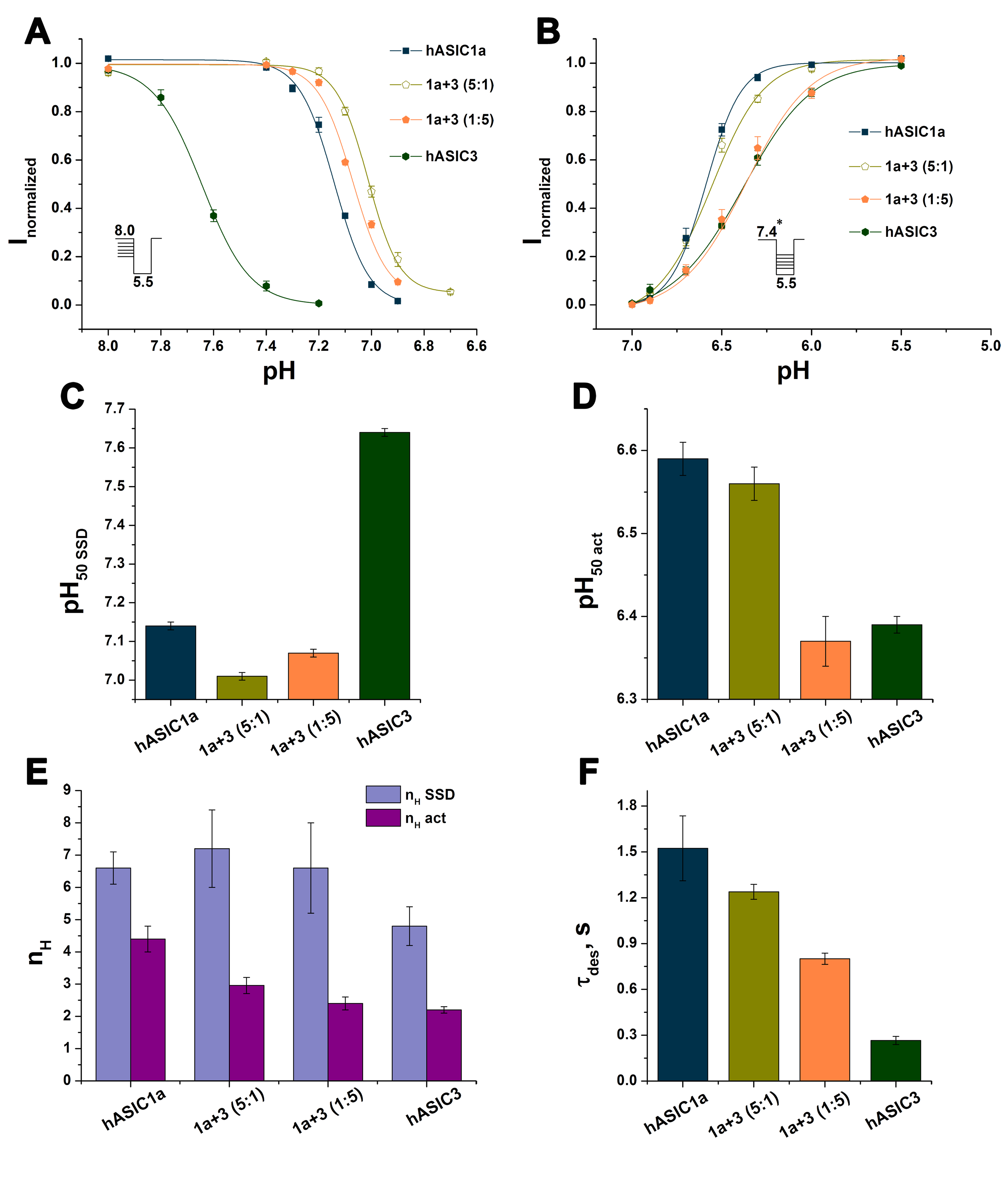


**Figure S2. Characterization of pH dependence of SSD and activation for hASIC1a, hASIC3, and 1a+3 heteromers.** (**A, B**) pH dependence of steady-state desensitization (**A**) and activation (**B**), fitted with Hill function (solid lines). In panel B, the asterisk indicates that pH dependence of activation was determined for all channels at a conditioning pH of 7.4, except for hASIC3, where the conditioning pH was 8.0. Each point represents data from 5-6 cells. (**C, D**) Bar plots of the half-maximal pH values obtained for SSD (pH₅₀ SSD) (**C**) and activation (pH₅₀ act) (**D**) of the channels. (**E**) Hill coefficients (n_H_) for SSD and activation. (**F**) Time constants of exponential decay (τ_des_) for currents of the corresponding channels, measured at a conditioning pH of 7.4 and activating pH of 5.5, except for hASIC3, measured at pH 8.0 and the activating pH 5.5. Data are presented as mean ± SEM.


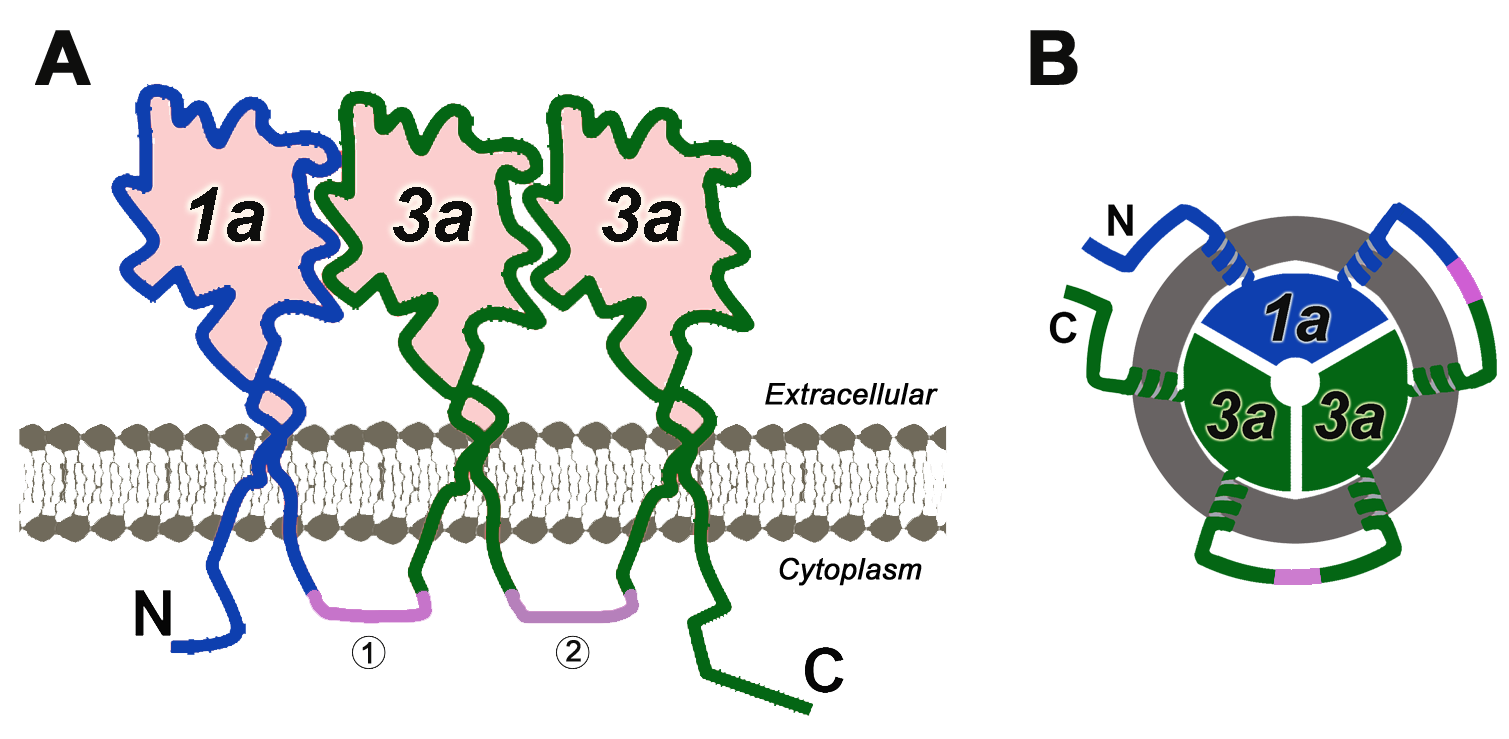


**Figure S3. Schematic representation of the concatemeric channel, using 1a-3-3 as an example.** (**A**) Side view of the concatemer. "1" and "2" represent linker 1 (NNVDINN) and linker 2 (NNQALNN), respectively. (**B**) Bottom view of the concatemer, with the lipid bilayer depicted in gray. "1a" corresponds to the human ASIC1a subunit, and "3a" corresponds to the human ASIC3 subunit. "N" denotes the amino terminus, while "C" denotes the carboxy terminus.


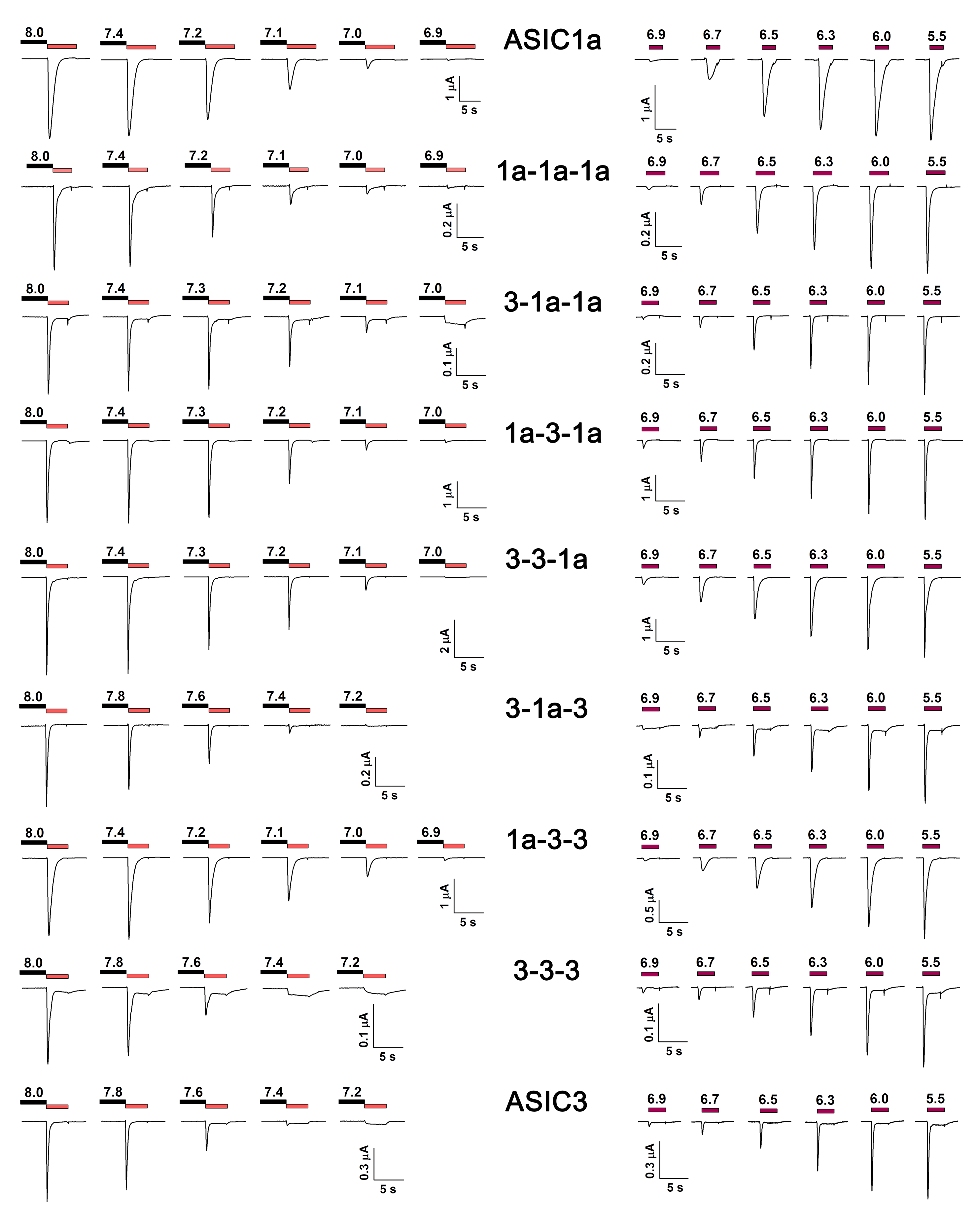


**Figure S4. pH dependence of steady-state desensitization (SSD) and activation (Act) for hASIC1a, hASIC3, and ASIC1a-3 concatemers.** Representative current traces for SSD (left panel) were recorded from the same cell, demonstrating activation by a pH 5.5 stimulus (indicated by the red bar) following pre-incubation at various conditioning pH levels. Activation traces (right panel) were recorded from the same cell, initiated from a conditioning pH of 7.4, except for ASIC3, 3-3-3, and 3-1a-3, which were conditioned at pH 8.0, followed by stimulation with different pH values.


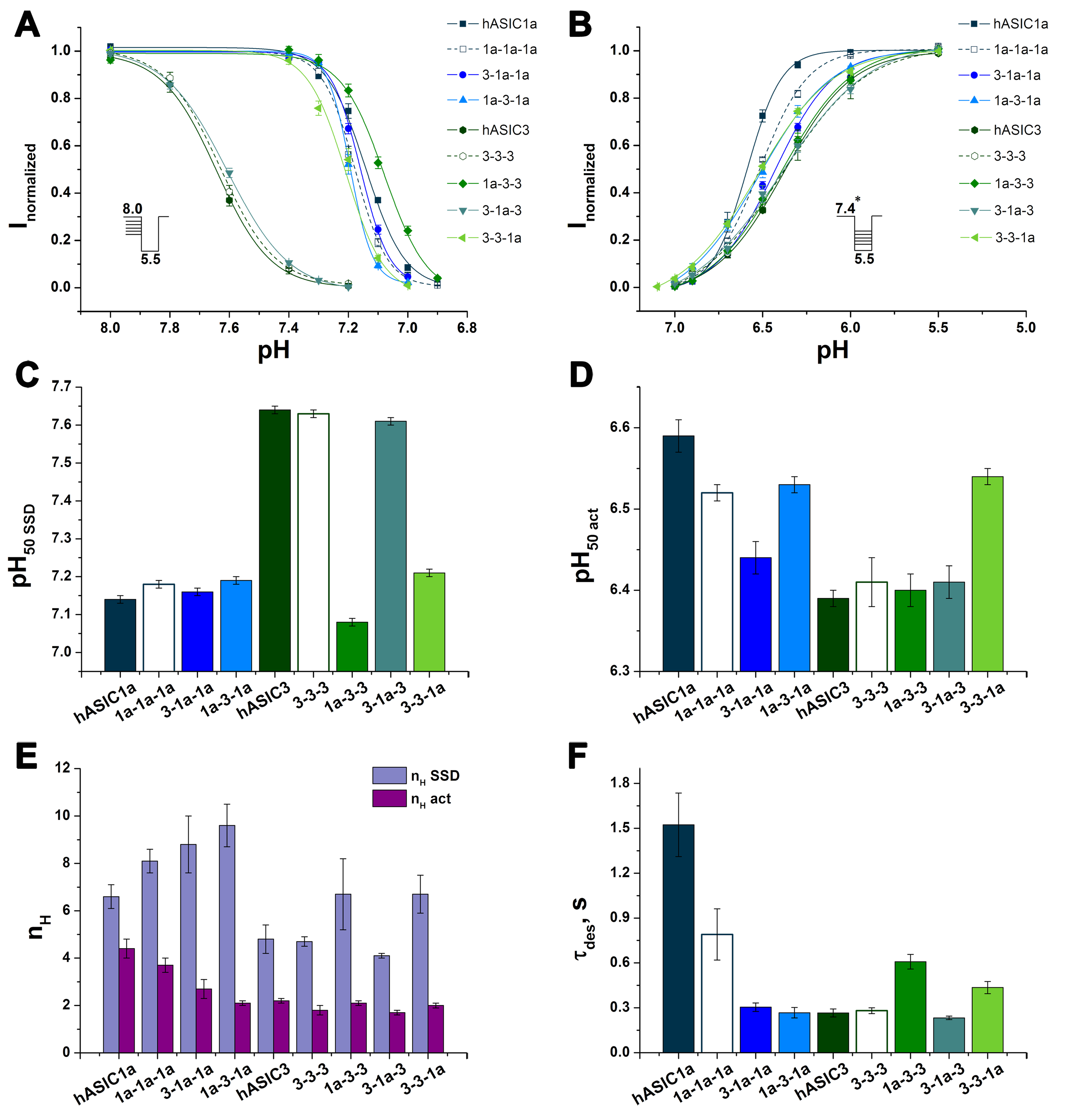


**Figure S5. Characterization of pH dependence of SSD and activation for hASIC1a, hASIC3, and 1a-3 concatemers.** (**A, B**) pH dependence of SSD (**A**) and activation (**B**), with data fitted using Hill function (solid lines). In panel B, the asterisk denotes that the pH dependence of activation was assessed at a conditioning pH of 7.4 for all channels, except for hASIC3, 3-3-3, and 3-1a-3, which were conditioned at pH 8.0. Each point represents data from 5-9 cells. (**C, D**) Bar plots showing the pH₅₀ for SSD (**C**) and activation (**D**) across the different channels. (**E**) n_H_ values for SSD and activation. (**F**) τ_des_ values for the corresponding channels, measured at a conditioning pH of 7.4 and an activating pH of 5.5, except for hASIC3, 3-3-3, and 3-1a-3, which were measured at a conditioning pH of 8.0 and an activating pH of 5.5. Data are presented as mean ± SEM.


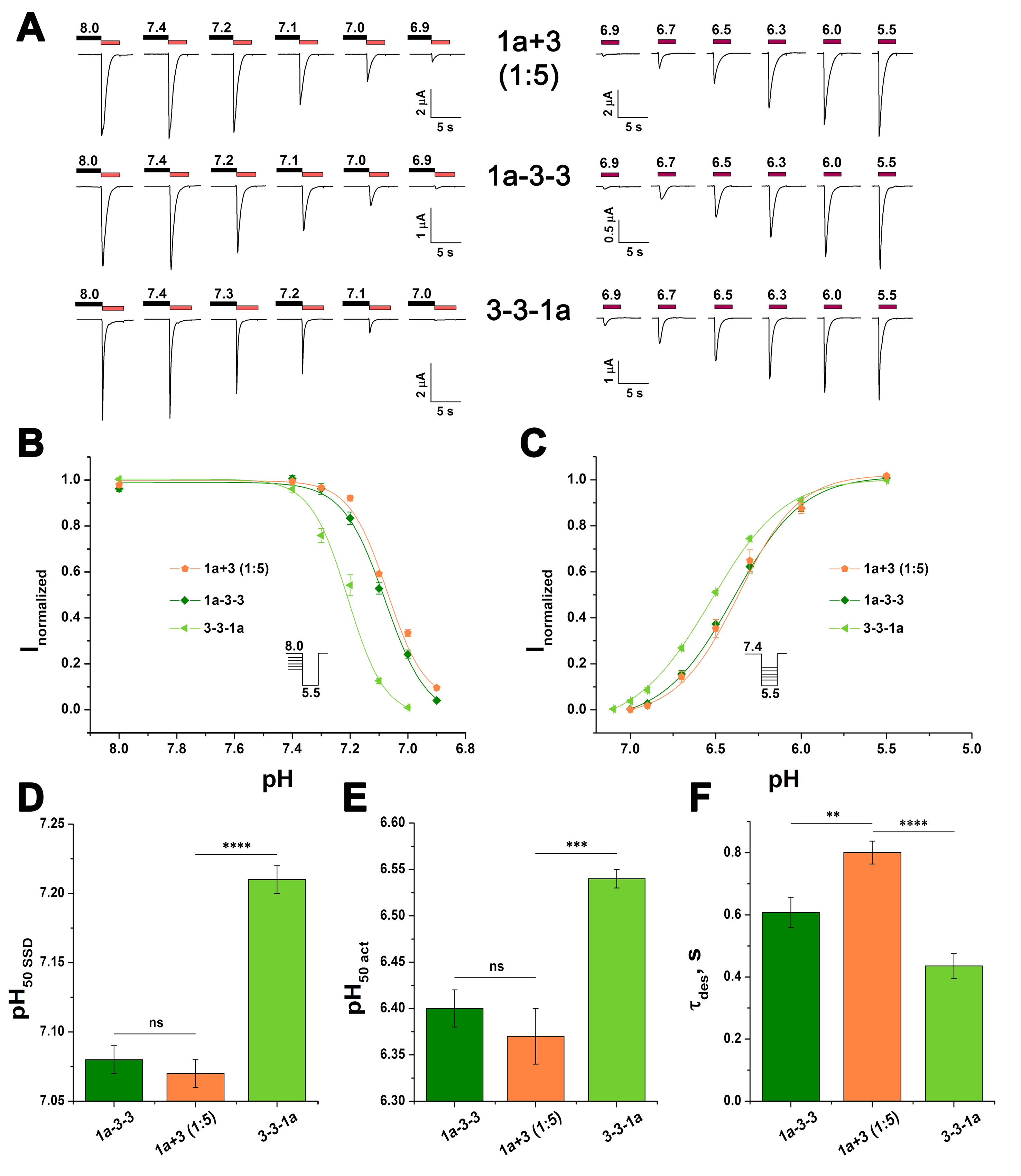


**Figure S6. The heteromeric 1a+3 (1:5) channel and the concatemeric 1a-3-3 channel exhibit similar functional characteristics.** (**A**) Representative current traces for steady-state desensitization (SSD, left panel) and activation (right panel) of the indicated channels. In the left panel, the red bar marks the application of pH 5.5 as a stimulus. (**B, C**) pH dependence curves for SSD (**B**) and activation (**C**) for the same channels. (**D-F**) Bar plots representing the pH₅₀ for SSD (**D**), activation (**E**), and τ_des_ (**F**) values for each channel type. Data are presented as mean ± SEM. Statistical significance: **p < 0.01, ***p < 0.001, ******p < 0.0001; ns indicates not significant (unpaired t-test).**


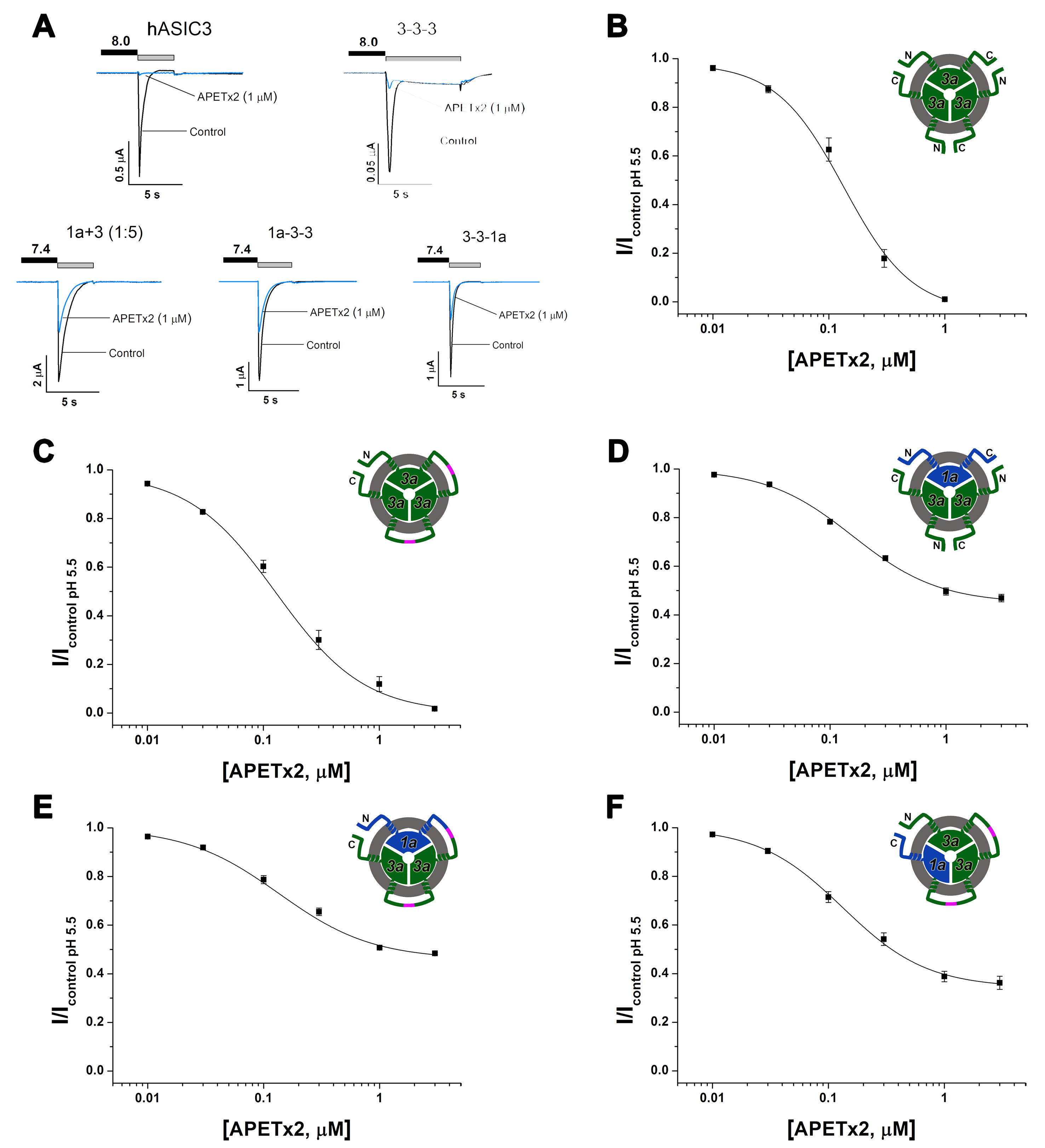


**Figure S7. The peptide inhibitor APETx2 exhibits similar effects on the 1a+3 (1:5) heteromer and the 1a-3-3 concatemer.** (**A**) Representative current traces showing the effect of 1 µM APETx2 on wild-type human ASIC3 (hASIC3), the 1a+3 (1:5) heteromer, and the concatemeric channels 3-3-3, 1a-3-3, and 3-3-1a. APETx2 was preincubated for 30 s, followed by activation with a pH 5.5 stimulus. (**B-F**) Dose–response curves for the inhibitory effect of APETx2 on hASIC3 (n = 5) (**B**), 3-3-3 (n = 5) (**C**), 1a+3 (1:5) (n = 10) (**D**), 1a-3-3 (n = 5) (**E**), and 3-3-1a (n = 9) (**F**). Data are presented as mean ± SEM.

## Table S1. Characteristics of hASIC1a and hASIC3 channels, concatemers of their subunits and hASIC1a and hASIC3 co-expressing at different mRNA ratios.

| **Channel** | **SSD** | | | **Act** | | | **τ_des_, s** |
| --- | --- | --- | --- | --- | --- | --- | --- |
|  | **pH_50_** | **Hill** | **n** | **pH_50_** | **Hill** | **n** |  |
| hASIC1a | 7.14 ± 0.01 | 6.6 ± 0.5 | 6 | 6.59 ± 0.02 | 4.4 ± 0.4 | 6 | 1.52 ± 0.21 |
| 1a-1a-1a | 7.18 ± 0.01 | 8.1 ± 0.5 | 9 | 6.52 ± 0.01 | 3.7 ± 0.3 | 7 | 0.79 ± 0.17 |
| 3-1a-1a | 7.16 ± 0.01 | 8.8 ± 1.2 | 7 | 6.44 ± 0.02 | 2.7 ± 0.4 | 5 | 0.30 ± 0.03 |
| 1a-3-1a | 7.19 ± 0.01 | 9.6 ± 0.9 | 5 | 6.53 ± 0.01 | 2.1 ± 0.1 | 5 | 0.27 ± 0.03 |
| hASIC3 | 7.64 ± 0.01 | 4.8 ± 0.6 | 5 | *6.39 ± 0.01 | 2.2 ± 0.1 | 5 | *0.27 ± 0.03 |
| 3-3-3 | 7.63 ± 0.01 | 4.7 ± 0.2 | 5 | *6.41 ± 0.03 | 1.8 ± 0.2 | 5 | *0.28 ± 0.02 |
| 1a-3-3 | **7.08 ± 0.01** | **6.7 ± 1.5** | 6 | **6.4 ± 0.02** | **2.1 ± 0.1** | 6 | 0.61 ± 0.05 |
| 3-1a-3 | 7.61 ± 0.01 | 4.1 ± 0.1 | 6 | *6.41 ± 0.02 | 1.7 ± 0.1 | 6 | *0.23 ± 0.01 |
| 3-3-1a | 7.21 ± 0.01 | 6.7 ± 0.8 | 6 | 6.54 ± 0.01 | 2.0 ± 0.1 | 5 | 0.44 ± 0.04 |
| 1a+3 (1:5) | **7.07 ± 0.01** | **6.6 ± 1.4** | 6 | **6.37 ± 0.03** | **2.4 ± 0.2** | 6 | 0.80 ± 0.04 |
| 1a+3 (5:1) | 7.01 ± 0.01 | 7.2 ± 1.2 | 6 | 6.56 ± 0.02 | 2.9 ± 0.3 | 6 | 1.24 ± 0.05 |

SSD – steady-state desensitization; Act – activation; n – number of independent oocytes; τ_des_ – time constant of exponential decay at pH 5.5; * - data obtained at conditioning pH 8.0 instead of 7.4. Data are presented as mean ± SEM.
